# Kinetics of the Neutralizing and Spike SARS-CoV-2 Antibodies following the Sinovac Inactivated Virus Vaccine Compared to the Pfizer mRNA Vaccine in Singapore

**DOI:** 10.3390/antib11020038

**Published:** 2022-05-27

**Authors:** Chin Shern Lau, May Lin Helen Oh, Soon Kieng Phua, Ya Li Liang, Yanfeng Li, Jianxin Huo, Yuhan Huang, Biyan Zhang, Shengli Xu, Tar Choon Aw

**Affiliations:** 1 Department of Laboratory Medicine, Changi General Hospital, Singapore 529889, Singapore; soon_kieng_phua@cgh.com.sg (S.K.P.); yali_liang@cgh.com.sg (Y.L.L.); aw.tar.choon@singhealth.com.sg (T.C.A.); 2 Department of Infectious Diseases, Changi General Hospital, Singapore 529889, Singapore; helen.oh.m.l@singhealth.com.sg; 3 GenScript Biotech (Singapore) Pte Ltd., Singapore 349248, Singapore; yanfeng.li@genscript.com; 4 Singapore Immunology Network, Agency for Science, Technology and Research, Singapore 138648, Singapore; huo_jianxin@immunol.a-star.edu.sg (J.H.); huang_yuhan@immunol.a-star.edu.sg (Y.H.); zhang_biyan@immunol.a-star.edu.sg (B.Z.); xu_shengli@immunol.a-star.edu.sg (S.X.); 5 Department of Physiology, Yong Loo Lin School of Medicine, National University of Singapore, Singapore 117599, Singapore; 6 Department of Medicine, National University of Singapore, Singapore 117599, Singapore; 7 Academic Pathology Program, Duke-NUS Medical School, Singapore 169857, Singapore

**Keywords:** mRNA vaccine, inactivated virus vaccine, antibodies, SARS-CoV-2

## Abstract

Introduction: We compared the early total spike antibody (S-Ab) and neutralizing antibody (N-Ab) responses to two vaccines. Methods: We studied 96 Pfizer and 34 Sinovac vaccinees over a 14-month period from January 2021 to February 2022. All vaccinees received three doses of one type of vaccine. Antibody levels (Roche Elecsys total S-Ab and the Snibe N-Ab) were tested 10 days after the first dose, 20 days after the second dose, and 20 days after the booster dose. Results: At all time points, the mRNA vaccine generated higher S-Ab and N-Ab responses than the inactivated virus vaccine (S-Ab: first dose 2.48 vs. 0.4 BAU/mL, second dose 2174 vs. 98 BAU/mL, third dose 15,004 vs. 525 BAU/mL; N-Ab: first dose 0.05 vs. 0.02 µg/mL, second dose 3.48 vs. 0.38 µg/mL, third dose 19.8 vs. 0.89 µg/mL). mRNA vaccine recipients had a 6.2/22.2/28.6-fold higher S-Ab and 2.5/9.2/22.2-fold higher N-Ab response than inactivated virus vaccine recipients after the first/second/third inoculations, respectively. Mann–Whitney U analysis confirmed the significant difference in S-Ab and N-Ab titers between vaccination groups at each time point. Conclusions: The mRNA vaccines generated a more robust S-Ab and N-Ab response than the inactivated virus vaccine at all time points after the first, second, and third vaccinations.

## 1. Introduction

The widespread implementation of vaccination programs has helped to reduce the burden of the current SARS-CoV-2 pandemic across the world. However, different vaccines have been employed in different countries. Two of the most widely available and utilized vaccines are the mRNA and inactivated virus vaccines. The evidence for the efficacy of mRNA vaccines in systemic reviews is robust [1], with trials showing 94.1% efficacy for the mRNA-1273 vaccine (Moderna) in preventing COVID-19 and 95% effectiveness with the BNT162b2 (Pfizer) vaccine in systemic reviews. There are some reports of breakthrough infections after a booster dose of the mRNA vaccine [2]. Thus, some have begun calling for a fourth dose of mRNA vaccine to better fight against new SARS-CoV-2 variants, such as the recent Omicron SARS-CoV-2 variant. In a recent study [3], 154/120 subjects received a fourth dose of the BNT162b2/mRNA-1273 vaccine, resulting in a 9–10-fold increase in anti-SARS-CoV-2 IgG antibodies and a 10-fold increase in anti-Omicron neutralization antibody titers. mRNA vaccines might become even more prevalent in the prevention of other diseases besides COVID-19 [4].

However, the situation is quite different with the inactivated virus vaccines (e.g., Sinovac). Several studies have questioned the efficacy of the Sinovac vaccine. In 11,303 volunteers aged between 18–59 years, the inactivated virus vaccine showed only 83.5% efficacy for the prevention of symptomatic COVID-19 after two doses (when compared to a placebo group), with nine cases of breakthrough COVID-19 infections in the vaccinated group (*n* = 6646) over a follow-up period of 43 days [5]. However, in a larger national cohort study (approximately 10.2 million subjects) [6], the inactivated virus vaccine (CoronaVac) only demonstrated an effectiveness of 65.9% for the prevention of COVID-19 illness, with somewhat better efficacy in preventing hospitalization (87.5%), intensive care unit admission (90.3%), and mortality (86.3%). In another national study [7], individuals who received inactivated virus vaccines had a greater risk of being infected (adjusted incidence rate ratio 2.99) or developing severe disease (adjusted incidence rate ratio 4.80), while individuals vaccinated with the mRNA-1273 vaccine were at lower risk of severe disease (adjusted incidence rate ratio 0.45). In a systemic review comparing seven SARS-CoV-2 vaccines [8], BNT162b2 (Pfizer) and mRNA-1273 (Moderna) vaccines had the highest efficacy in preventing symptomatic COVID-19 (P-scores 0.952 and 0.843), while the inactivated virus vaccine (CoronaVac) had a lower efficacy (P-score 0.570). Another systemic review [9] showed that two mRNA vaccine doses conferred a lesser risk of SARS-CoV-2 infection (odds ratio: 0.05; 95% confidence interval: 0.02–0.13) than vaccination with viral vector and inactivated vaccines. Inactivated virus vaccines may also carry a greater risk of post-vaccination breakthrough infection than mRNA vaccines [10]. In this study, in 336 post-vaccine breakthrough infections, the hazard ratio for breakthrough infections in mRNA vaccinees was only 0.58 compared to the inactivated virus vaccine group, with mRNA vaccinees having a longer time to breakthrough after completion of two vaccine doses (111.5 vs. 81 days).

The reduced efficacy of the inactivated viral vaccine may be attributed to lower SARS-CoV-2 antibody response to the vaccine. In one study [11] that compared the neutralization antibody response (surrogate virus neutralization and plaque reduction neutralization assays) between two doses of BNT162b2 vs. CoronaVac vaccines, geometric mean PRNT_50_ titers were 269 (mRNA vaccine) vs. 27 (inactivated virus vaccine) after the second vaccine dose. Even in heterologous vaccination regimens, where a second dose of mRNA vaccine provided a 100% seropositivity 3 months post-vaccination, the CoronaVac vaccine could only generate a 60–76% seropositivity rate at that time point [12]. Furthermore, heterologous vaccination using mRNA vaccines also seems to be more efficacious against the new SARS-CoV-2 variants, generating higher Omicron-specific antibody geometric mean titer levels (27.6 vs. 5.83) in patients who previously received two doses of inactivated virus vaccine (23.8) [13].

In our country, the majority of the population was vaccinated with the Pfizer BNT162b2 mRNA vaccine. However, some individuals opted to receive the Sinovac inactivated virus vaccine for medical and personal reasons. There is no information on the antibody response to inactivated virus vaccines in Singapore, and how it compares to the mRNA vaccines. We, thus, compared the early total spike antibody (S-Ab) and neutralizing antibody (N-Ab) responses to each of these two vaccines in our local population.

## 2. Methods

### 2.1. Study Participants

We studied 96 Pfizer vaccinees (28 males, 68 females, mean age 42.3 ± 13.4 years) and 34 Sinovac vaccinees (11 males, 23 females, mean age 46.1 ± 13.3 years) over a 14-month period from January 2021 to February 2022, during which our country experienced two waves of COVID-19 infections (one of the Delta variant in the last quarter of 2021, one of the Omicron variant at the start of 2022). All participants had no prior history or reported COVID-19 infections during the study period and were not immunosuppressed. At baseline, all subjects were not previously exposed to COVID-19, as evidenced by negative nucleocapsid SARS-CoV-2 antibodies pre-vaccination. All vaccinees received either three homologous doses of mRNA vaccine or inactivated virus vaccine. Antibody levels were tested 10 days after the first dose, 20 days after the second dose, and 20 days after the booster dose. Due to different vaccination schedules, the number of samples at each time point was different. All samples obtained in this study were de-identified and anonymized.

### 2.2. Methods and Materials

Serum at each time point was obtained and stored at −70 degrees Celsius if not immediately analyzed. Frozen samples were thawed for 1 h at room temperature just prior to analysis. Thawed samples were vortexed before analysis. The Roche Elecsys Anti-SARS-CoV-2 S assay (a quantitative double-antigen sandwich electro-chemiluminescent immunoassay performed on the Roche Elecsys e801 auto-analyzer) and the Snibe competitive quantitative N-Ab assay (performed on the Snibe Maglumi) have been previously described and utilized in prior studies from our laboratory [14,15]. The Roche total S-Ab assay has a positive threshold of ≥0.78 BAU/mL (manufacturer claimed assay upper limit 243 BAU/mL, dilution range up to 1:100, limit of detection 0.31 BAU/mL, reported precision of 2.9% and 1.4% at 0.47 and 178 BAU/mL; reported assay sensitivity of 98.8% and specificity of 99.98%). In the Snibe N-Ab assay, sample N-Ab competes with angiotensin-converting enzyme 2 antigen immobilized on a solid phase for binding labelled recombinant SARS-CoV-2 S Receptor Binding Domain antigen to produce a light signal that is inversely proportional to the sample N-Ab. The Snibe assay has a positive threshold of ≥0.3 μg/mL (manufacturer claimed measuring range of 0.05–30 μg/mL, claimed interassay precision is 1.27% and 1.01% at 0.079 and 21.192 μg/mL, respectively, limit of detection 0.045 μg/mL, sensitivity of 100%, and specificity 100%). Although the Roche total S-Ab assay reports titers in U/mL, this can be converted to WHO international units (BAU/mL = 0.97 × U/mL) [16].

### 2.3. Statistical Analysis

Data were presented as medians with ranges where appropriate. No indeterminate or missing results were used. We utilized the Mann–Whitney U test to compare the antibody titers between different vaccination groups at each time point, with *p* < 0.05 considered statistically significant. MedCalc Statistical Software (version 20.008, MedCalc Software Ltd., Ostend, Belgium) was utilized for statistical analyses. Our hospital’s Institutional Review Board deemed this work exempt as part of a seroprevalence survey using de-identified, anonymized samples/data. However, informed consent was still obtained from all subjects involved, as they needed to provide blood samples at the three time points. Compliance with STARD guidelines is enclosed (see Supplementary Table S1).

## 3. Results

### 3.1. Comparison of Responses between mRNA and Inactivated Virus Vaccines

**Figure 1:**
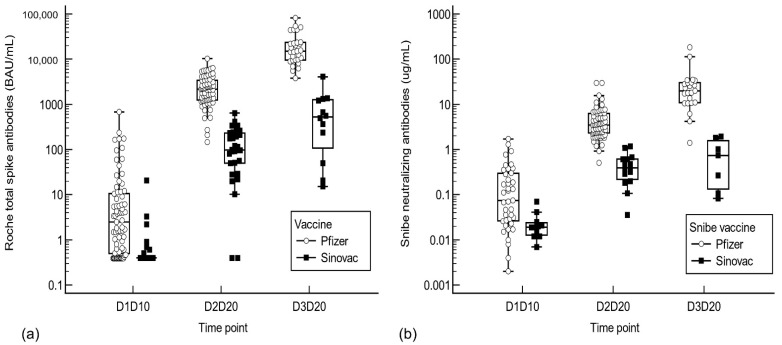
(**a**) Roche total spike and (**b**) Snibe neutralizing antibody responses after the first, second, and third dose of inactivated virus or mRNA vaccine. Abbreviations: D1D10: 10 days post-dose 1, D2D20: 20 days post-dose 2, D3D20: 20 days post-dose 3.

At every time point, the mRNA vaccine generated higher S-Ab and N-Ab responses than the inactivated virus vaccine (see Figure 1, Supplementary Table S2). mRNA vaccine recipients had a 6.2/22.2/28.6-fold higher S-Ab and 2.5/9.2/22.2-fold higher N-Ab response than inactivated virus vaccine recipients after the first/second/third inoculations, respectively. Mann–Whitney U analysis confirmed the significant difference in S-Ab and N-Ab titers between vaccination groups at each time point (see Table 1).

### 3.2. Comparison of the Rise in Antibodies between Doses

**Figure 2:**
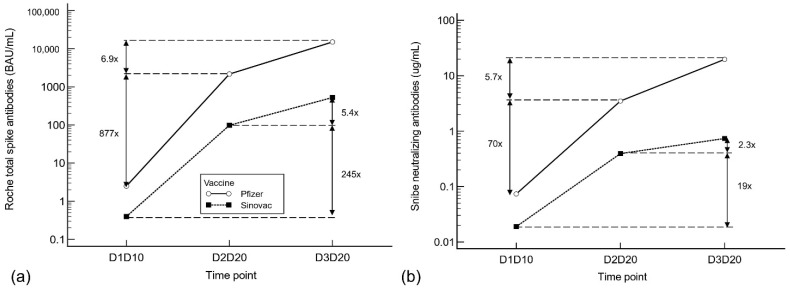
(**a**) Roche total S-Ab and (**b**) Snibe N-Ab trend at the three time points between the Pfizer and Sinovac vaccines, with the fold increase between each point. Abbreviations: D1D10: 10 days post-dose 1, D2D20: 20 days post-dose 2, D3D20: 20 days post-dose 3.

We compared the median antibody responses between the first, second, and third vaccine inoculations. For the inactivated virus vaccine, the second dose increased the median S-Ab titer by 245-fold from the first dose, and the third dose increased the S-Ab titer by 5.4-fold from the second dose (see Figure 2). The mRNA vaccine generated a greater rise in antibody titers than the inactivated virus vaccine, with the median S-Ab titer rising by 877- and 6.9-fold after the second and third doses. This pattern was also reflected in the N-Ab titers, with the N-Ab increasing by 19-/2.3-fold after the second/third dose of inactivated virus vaccine, but increasing by 70-/5.7-fold after the two doses of mRNA vaccine.

### 3.3. Analysis by Gender Groups

**Figure 3:**
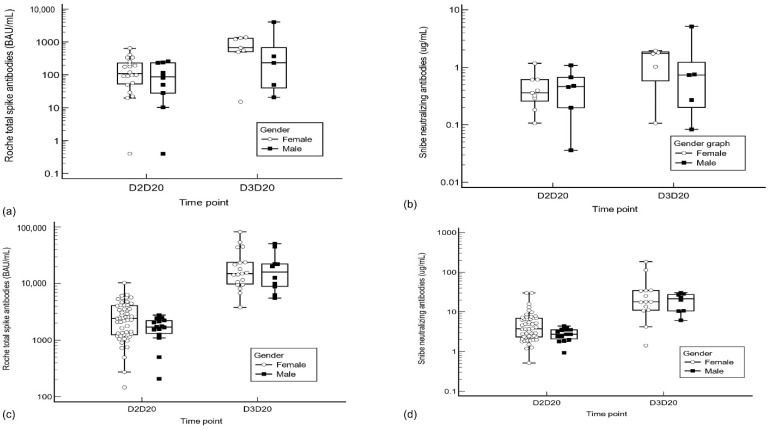
Gender group comparison of spike and neutralizing antibody responses between mRNA/inactivated virus vaccinees. (**a**) Roche responses in Sinovac vaccinees, (**b**) Snibe responses in Sinovac vaccinees, (**c**) Roche responses in Pfizer vaccinees, (**d**) Snibe responses in Pfizer vaccinees. Abbreviations: D2D20: 20 days post-dose 2, D3D20: 20 days post-dose 3.

We also compared the S-Ab and N-Ab responses after the second and third inoculation by gender groups. Mann–Whitney U testing showed no significant differences in S-Ab/N-Ab responses between male and female Pfizer/Sinovac vaccinees at all time points (see Figure 3, Supplementary Table S3). 

## 4. Discussion

We found that the early S-Ab and N-Ab antibody responses to the mRNA vaccine (Pfizer) were significantly higher than those elicited by the inactivated virus vaccine (Sinovac) at all time points after the first, second, and booster doses, with a greater fold rise in antibodies after the second and third mRNA vaccine inoculations. This finding is also supported by other studies. In 457 healthcare workers in Hong Kong, the IgG (Abbott) and total (Roche) S-Ab levels induced by the BNT162b2 mRNA vaccine were higher for both dose 1 and dose 2 than those induced by CoronaVac inactivated virus vaccine: after dose 1 (CoronaVac vs. BNT162b2), total S-Ab 9.6 vs. 104.2 U/mL, IgG S-Ab 158 vs. 1618 AU/mL; after dose 2, total S-Ab 142 vs. 244 U/mL, IgG S-Ab 1005 vs. 11573 AU/mL. In addition, more BNT162b2 recipients had positive surrogate N-Abs (GenScript cPass) after dose 2 of the mRNA vaccine compared to the inactivated virus vaccine (100% vs. 94%; *p* < 0.0194) [17]. In another study of 288 participants [18], 99.3% of mRNA vaccine recipients were positive for IgG (VIDAS, Biomerieux Inc., Hazelwood, MO, USA) 6 weeks after the second dose of the vaccine, with mean titers of 515.5 BAU/mL, but only 85.7% of inactivated virus vaccine recipients were seropositive, with lower mean S-Ab titers around three-fold less than the titers of the mRNA vaccine group (170 BAU/mL). Even in patients with previous COVID-19 infection, a post-infection booster of mRNA vaccine (BNT162b2) caused 20.4–22.3-fold increases in virus neutralization titers, while the inactivated vaccine only resulted in a 1.8–2.2-fold increase [19]. Our findings are also supported by studies of patients who received heterologous dosing regimens [20], where a third dose of mRNA vaccine in patients who already received two doses of inactivated virus vaccine elicited a much more robust anti-receptor binding domain antibodies 14 days after inoculation (third dose inactivated virus vaccine: antibody geometric mean titer 1073 U/mL, third dose mRNA vaccine: antibody geometric mean titer 20,787 U/mL).

Our study focused on time points that approximate the peak antibody levels that would develop in response to vaccination. We previously demonstrated that peak total S-Ab and N-Ab responses were 20/30 days after the second/booster doses of the Pfizer vaccine [14,15]. Other studies demonstrate peak IgG and total S-Ab responses 1 week after the second dose of the BNT162b2 vaccine [21,22]. Another study [23] showed that peak SARS-CoV-2 neutralizing antibodies (pseudovirus neutralization assay) was maintained for up to 30 days post-second vaccination, with antibodies declining with each month only after 30 days. Thus, the early antibody responses reported in our study should be a close approximate of the peak antibody titers elicited by each vaccine.

The lower antibody titers elicited by inactivated virus vaccines would be of even greater clinical concern for certain vulnerable groups of patients. For example, in immunosuppressed kidney transplant patients, the response to the different types of vaccine also showed a similar pattern, with 36.5%/27.8% of patients seroconverting post-mRNA/inactivated virus vaccination, with greater post-mRNA vaccination antibody levels (median 173 (mRNA) and 29 (inactivated virus) BAU/mL, *p* < 0.034) [24]. Similarly, in a study examining multiple sclerosis patients treated with disease modifying therapy, the inactivated vaccine only resulted in a seroconversion rate of 64.9%, much less than the 81.2% seroconversion rate of the mRNA vaccine [25]. Although there is no precise level of S-Ab or N-Ab that is considered definitively protective, several studies have shown that higher levels of antibodies correlate well with protection against severe SARS-CoV-2 infection [26,27,28]. Indeed, in the Bergwerk study of breakthrough infections in vaccinated healthcare workers [26], peri-infection geometric mean titers of neutralizing antibodies (pseudovirus neutralization assay) were 193/530 in cases/controls, and geometric mean titer of IgG (Beckman Coulter) was 11.2/21.8 between cases and controls. Thus, the lower antibody titers in immunocompromised/immunosuppressed patients following inactivated virus vaccines would place them at greater risk for COVID-19 breakthrough infection. On the other hand, with two doses of BNT162b2/mRNA-1273 mRNA vaccines, even immunocompromised patients with solid cancers, hematologic cancers, autoimmune diseases, or solid organ transplants could develop high enough rates of seropositivity one month after the second vaccine inoculation (100% of subjects with untreated solid cancers, 98.3% of those with treated solid cancers, and 95% of those with untreated hematologic cancers), conferring some degree of protection against COVID-19 [29].

Although all antibody titers wane with time, some studies report that antibodies generated by the inactivated virus vaccine decline faster than those formed in response to the mRNA vaccine. In an evaluation [30] of 850 healthy blood donors’ IgG S-Ab (in-house ELISA) and N-Ab (surrogate virus neutralization test, GenScript), both median S-Ab and N-Ab levels dropped below assay cut-off in inactivated virus vaccinees by the third and fourth months post-second dose respectively, with only 26.3%/21.1% of vaccinees remaining seropositive 4 months after the second inoculation. On the other hand, mRNA vaccinees retained 97.1% IgG S-Ab and N-Ab positivity at these time points. To further compound the issue, studies have also shown that the inactivated virus vaccines may not provide adequate neutralization against several prevalent variants of SARS-CoV-2. When assessing neutralizing SARS-CoV-2 antibodies on a virus cell culture assay (assessed in geometric mean titers), lower neutralization titers were found to the alpha and gamma variants of SARS-CoV-2 (18.5 and 10.0) even after two doses of inactivated virus vaccine [31]. However, even in patients with co-morbidities such as untreated/treated solid cancers, mRNA vaccines elicited N-Ab titers to Alpha, Beta, Gamma, and Delta variants of SARS-CoV-2 that were only slightly lower than healthy controls 1 month after vaccination (up to 98.3% seropositivity in untreated cancer and 94.3% in treated solid cancers) [29].

We, thus, report the following novel findings:

➢ We have performed a head-to-head comparison of two vaccination strategies, using the same assays for two different antibodies, with one generating results in WHO units;
➢ In our local population, inoculation with the mRNA vaccine generated a quantitatively higher S-Ab and N-Ab titer than the inactivated virus vaccine after all three doses;
➢ The fold increase in S-Ab and N-Ab after each dose was higher with the mRNA vaccine than the inactivated virus vaccine.

Our findings, as well as those of these other studies, demonstrate certain drawbacks with the use of inactivated virus vaccines. Thus, for patients who have already received two doses of inactivated virus vaccine, it may be prudent to consider a third booster dose of mRNA vaccine, particularly if they are ≥50 years old. Studies clearly show the benefits of such a heterologous vaccination regimen. In a study [32] of 560 healthcare workers, median IgG S-Ab (Abbott) decreased from 473.6 AU/mL to 166.5 AU/mL from the first and fourth month after the second dose of inactivated virus vaccine, with a drop in seropositivity from 98.9% to 89.1%. After a booster dose of the mRNA and inactivated virus vaccine, S-Ab levels increased by 104.8-fold and 8.7-fold, respectively, a 14.2-fold increase in favor of mRNA vaccination compared to the inactivated virus vaccine. Another study [33] compared the response of a third dose of Ad26.COV2-S/BNT162b2/ChAdOx1/CoronaVac vaccine in subjects with two prior doses of CoronaVac vaccine, and the results showed a 77-/152-/90-/12-fold rise in IgG S-Ab after the booster dose, with all heterologous booster regimens inducing higher concentrations of IgG S-Ab and pseudovirus neutralizing antibodies. Even 4 weeks after the booster dose, older adults (≥61 years old) still had 100% seropositivity with the mRNA vaccine booster.

A limitation of our study is that none of our population contracted COVID-19 during the study period. Thus, we were unable to assess whether the two vaccines conferred differing levels of protection from SARS-CoV-2 infection. The rates of breakthrough infection between the two vaccines could also not be ascertained. However, the aforementioned studies [7,8,9] have already established the increased clinical efficacy of the mRNA vaccines over the inactivated virus vaccines in the prevention of COVID-19 infections. Another limitation is the relatively small numbers of subjects that received the inactivated virus vaccine, as well as subjects who received a third dose of either mRNA/inactivated virus vaccine. We were unable to examine the different antibody responses in different age groups as there were too few subjects, and larger studies would be desirable. All Sinovac vaccinees were deemed COVID-19 naïve throughout the study based on the absence of symptomatic COVID-19 infection, as all subjects were SARS-CoV-2 nucleocapsid antibody negative at baseline. However, as nucleocapsid and S-Ab rose after inoculation with the inactivated vaccine, we cannot be certain about their exposure to COVID-19 post-vaccination. Inactivated virus vaccines target a range of antigens, including the nucleocapsid proteins of the virion, whereas mRNA vaccines are more specific in targeting the spike proteins [34]. Thus, inactivated vaccines do induce a rise in nucleocapsid antibodies [35] and prevents its use in the diagnosis of asymptomatic/pauci-symptomatic post-vaccination infection as well as the identification of previous exposure to COVID-19. The nucleocapsid antibodies developed in response to inactivated vaccines may have a theoretical benefit against the Omicron strain; however, real-world evidence [36] has shown that mRNA vaccines, even when used in a heterologous dosing regimen, provided greater protection against COVID-19 infection during waves of Omicron predominance. Although we were able to assess S-Ab and N-Ab responses to vaccines, we were unable to assess the development of memory B cells in response to inactivated vaccines. There is evidence that memory B cells develop in response to the mRNA vaccines [37], although how much they contribute to immunity is unclear.

## 5. Conclusions

The mRNA vaccines generated a more robust S-Ab and N-Ab response than the inactivated virus vaccine at all time points after the first, second, and third vaccinations. It may be important to consider using the mRNA vaccine over the inactivated virus vaccine in groups that would mount a poorer antibody response to the inactivated virus vaccine.

## Figures and Tables

**Table 1:** Mann–Whitney U analysis of the antibody titers of each vaccination group after the first, second, and third vaccination.

	Time Point	Median Difference (95% CI) (BAU/mL)
mRNA vs. inactivated virus vaccine: Roche total spike antibodies	10 days post-dose 1	1.41 (0.55 to 3.69), *p* = 0.0001
	20 days post-dose 2	2033 (1470 to 2386), *p* < 0.0001
	20 days post-dose 3	14362 (9501 to 21705), *p* < 0.0001
mRNA vs. inactivated virus vaccine: Snibe neutralizing antibodies	10 days post-dose 1	0.03 (0.01 to 0.10), *p* = 0.0006
	20 days post-dose 2	2.92 (2.13 to 4.09), *p* < 0.0001
	20 days post-dose 3	18.4 (10.5 to 28.4), *p* < 0.0001

Abbreviations: CI: Confidence interval.

## Data Availability

All related data and methods are presented in this paper. Additional inquiries should be addressed to the corresponding author.

## Supplementary Materials

The following supporting information can be downloaded at: https://www.mdpi.com/article/10.3390/antib11020038/s1, Supplementary Table S1: STARD checklist, Supplementary Table S2: Quantitative values of Roche total spike and Snibe neutralizing antibody responses after the first, second, and third dose of inactivated virus or mRNA vaccine, Supplementary Table S3: Gender group comparison of spike and neutralizing antibody responses between mRNA/inactivated virus vaccinees.

## Abbreviations

S-Ab	Spike antibodies
N-Ab	Neutralizing antibodies
