# Supplementary material for: Kinetics of the Neutralizing and Spike SARS-CoV-2 Antibodies following the Sinovac Inactivated Virus Vaccine Compared to the Pfizer mRNA Vaccine in Singapore
Source: Antibodies (Basel). 2022 May 27;11(2):38. doi: 10.3390/antib11020038 (PMC9220327; doi:10.3390/antib11020038)
Supplement: Supplementary file 1 [file antibodies-11-00038-s001.zip › antibodies-1681252-supplementary.pdf]

Supplementary Table S1: STARD checklist

Section & Topic	No	Item	Reported on page #
**TITLE OR ABSTRACT**			
	**1**	Identification as a study of diagnostic accuracy using at least one measure of accuracy (such as sensitivity, specificity, predictive values, or AUC)	**2**
**ABSTRACT**			
	**2**	Structured summary of study design, methods, results, and conclusions (for specific guidance, see STARD for Abstracts)	**2**
**INTRODUCTION**			
	**3**	Scientific and clinical background, including the intended use and clinical role of the index test	**3**
	**4**	Study objectives and hypotheses	**4**
**METHODS**			
Study design	**5**	Whether data collection was planned before the index test and reference standard<br>were performed (prospective study) or after (retrospective study)	**5**
Participants	**6**	Eligibility criteria	**5**
	**7**	On what basis potentially eligible participants were identified (such as symptoms, results from previous tests, inclusion in registry)	**5**
	**8**	Where and when potentially eligible participants were identified (setting, location and dates)	**5**
	**9**	Whether participants formed a consecutive, random or convenience series	**5**
Test methods	**10a**	Index test, in sufficient detail to allow replication	**5**
	**10b**	Reference standard, in sufficient detail to allow replication	**5**
	**11**	Rationale for choosing the reference standard (if alternatives exist)	**5**
	**12a**	Definition of and rationale for test positivity cut-offs or result categories of the index test, distinguishing pre-specified from exploratory	**5**
	**12b**	Definition of and rationale for test positivity cut-offs or result categories of the reference standard, distinguishing pre-specified from exploratory	**5**
	**13a**	Whether clinical information and reference standard results were available to the performers/readers of the index test	**5**
	**13b**	Whether clinical information and index test results were available to the assessors of the reference standard	**5**
Analysis	**14**	Methods for estimating or comparing measures of diagnostic accuracy	**6**
	**15**	How indeterminate index test or reference standard results were handled	**6**
	**16**	How missing data on the index test and reference standard were handled	**6**
	**17**	Any analyses of variability in diagnostic accuracy, distinguishing pre-specified from exploratory	**6**
	**18**	Intended sample size and how it was determined	**Not applicable**
**RESULTS**			
Participants	**19**	Flow of participants, using a diagram	**Not applicable**
	**20**	Baseline demographic and clinical characteristics of participants	**5**
	**21a**	Distribution of severity of disease in those with the target condition	**Not applicable**
	**21b**	Distribution of alternative diagnoses in those without the target condition	**Not applicable**
	**22**	Time interval and any clinical interventions between index test and reference standard	**Not applicable**
Test results	**23**	Cross tabulation of the index test results (or their distribution) by the results of the reference standard	**6**
	**24**	Estimates of diagnostic accuracy and their precision (such as 95% confidence intervals)	**Not applicable**

	**25**	Any adverse events from performing the index test or the reference standard	**Not applicable**
**DISCUSSION**			
	**26**	Study limitations, including sources of potential bias, statistical uncertainty, and generalisability	**14**
	**27**	Implications for practice, including the intended use and clinical role of the index test	**13**
**OTHER INFORMATION**			
	**28**	Registration number and name of registry	**6**
	**29**	Where the full study protocol can be accessed	**Not applicable**
	**30**	Sources of funding and other support; role of funders	**Not applicable**

Supplementary Table S2. Quantitative values of Roche total spike and Snibe neutralizing antibody responses after the first, second and third dose of inactivated virus or mRNA vaccine.

	**Roche Total Spike Antibodies**				**Snibe Neutralizing Antibodies**			
	**Time Point**	**n**	**Median (95% CI) (BAU/mL)**	**Range (BAU/mL)**	**Time Point**	**n**	**Median (95% CI) (µg/mL)**	**Range (µg/mL)**
Inactivated virus vaccine	10 days post dose 1	30	0.4 (0.4-0.4)	0.4 – 20.6	10 days post dose 1	15	0.02 (0.002-0.02)	0 – 0.07
	20 days post dose 2	30	98.0 (60.6-190)	0.4 – 641	20 days post dose 2	16	0.38 (0.19-0.61)	0 – 1.17
	20 days post dose 3	12	525 (81.4-1301)	15.2 – 4067	20 days post dose 3	10	0.89 (0.18-1.89)	0.08 – 5.14
mRNA vaccine	10 days post dose 1	73	2.48 (1.38-4.16)	0.39 – 677	10 days post dose 1	73	0.05 (0.03-0.10)	0 – 1.71
	20 days post dose 2	68	2174 (1649-2484)	146 – 10363	20 days post dose 2	66	3.48 (2.71-3.99)	0.51 – 30.0
	20 days post dose 3	29	15004 (10452-22114)	3803-82203	20 days post dose 3	23	19.8 (11.9-29.4)	1.41 – 185

Abbreviations: CI: Confidence interval.

Supplementary Table S3. Gender group comparison of spike and neutralizing antibody responses between mRNA/inactivated virus vaccinees.

		**Sinovac vaccinees**		**Pfizer vaccinees**	
Roche Total Spike Antibodies	Time Point	20 days post dose 2	20 days post dose 3	20 days post dose 2	20 days post dose 3
	Females	20	7	53	19
	Median (BAU/mL)	110	670	2415	15004
	Range (BAU/mL)	0.4-641	15.2-1379	146-10363	3803-82203
	Males	10	5	15	10
	Median (BAU/mL)	87.9	234	1706	16433
	Range (BAU/mL)	0.4-259	20.8-4067	208-2772	5554-50595
	Difference (95% CI, p)	34.1 (-49.9 to 129, p=0.37)	446 (-2747 to 1159, p=0.29)	723 (-74.7 to 1689, p=0.08)	1283 (-7951 to 9382, p=0.78)
Snibe Neutralizing Antibodies	Time Point	20 days post dose 2	20 days post dose 3	20 days post dose 2	20 days post dose 3
	Females	10	5	51	14
	Median (μg/mL)	0.34	1.73	3.74	17.9
	Range (μg/mL)	0-1.17	0.11-1.95	0.51-30.0	1.41-184.5
	Males	6	5	15	8
	Median (μg/mL)	0.46	0.73	2.72	21.4
	Range (μg/mL)	0.04-1.08	0.08-5.14	0.92-4.38	6.09-30.4
	Difference (95% CI, p)	0.09 (-0.29 to 0.47, p=0.51)	0.94 (-3.42 to 1.68, p=0.35)	1.10 (-0.05 to 2.99, p=0.06)	3.17 (-10.3 to 15.2, p=0.63)

Abbreviations: CI: Confidence Interval.
